# Cloning, subcellular localization and expression of phosphate transporter gene *HvPT6* of hulless barley

**DOI:** 10.1515/biol-2022-0543

**Published:** 2023-05-08

**Authors:** Likun An, Xiaohua Yao, Youhua Yao, Yongmei Cui, Yixiong Bai, Xin Li, Kunlun Wu

**Affiliations:** College of Agriculture and Forestry Sciences, Qinghai University, Xining 810016, P.R. China; Qinghai Key Laboratory of Hulless Barley Genetics and Breeding, Xining 810016, P.R. China; Qinghai Subcenter of National Hulless Barley Improvement, Xining 810016, P.R. China

**Keywords:** hulless barley, phosphate transporter, *HvPT6* gene, bioinformatics analysis

## Abstract

Deficiency of phosphate (Pi) is one of the main growth-limiting factors for crops. Generally, phosphate transporters play a key role in the uptake of P in the crops. However, current knowledge regarding the molecular mechanism underlying Pi transport is still limited. In this study, a phosphate transporter (PT) gene, designated *HvPT6*, was isolated from a cDNA library constructed from hulless barley “Kunlun 14.” The promoter of HvPT6 showed a large number of elements related to plant hormones. The expression pattern also indicated that *HvPT*6 was highly induced by low phosphorus, drought, abscisic acid, methyl jasmonate and gibberellin. Phylogenetic tree analysis revealed that HvPT6 belongs to the same subfamily of the major facilitator superfamily as OsPT6 from *Oryza sativa*. Subcellular localization of HvPT6:GFP using transient expression of *Agrobacterium tumefaciens* showed the green fluorescent protein signal in the membrane and nucleus of the *Nicotiana benthamiana* leaves. Overexpressing HvPT6 led to a longer and higher lateral root length and dry matter yield in the transgenic *Arabidopsis* lines under low Pi conditions, indicating that HvPT6 improves plant tolerance under Pi-deficient conditions. This study will lay a molecular basis for phosphate absorption mechanism in barley and breeding barley with high-efficient phosphate uptake.

## 1 Introduction

Hulless barley (*Hordeum vulgare* L. var. *nudum* Hook. f.), a variant of barley, is one of the most important grain and forage crops in the Qinghai-Tibet Plateau China, and also is a symbol of agricultural civilization in the Qinghai-Tibet Plateau [1–3]. Due to long-term evolution and artificial cultivation, hulless barley has adapted to the extreme harsh climate such as cold, barren, drought and ultraviolet exposure in the Qinghai-Tibet Plateau [4–6]. It is the only crop that can grow well in this area at an altitude of 2,800 m. The production of hulless barley directly affects the food security and economic development of the Qinghai-Tibet Plateau [7,8].

Phosphorus (P) is one of the most important nutrients needed by the crops, and it has a direct effect on the growth and development, resistance, yield and quality of crops. Phosphorus in soil gets easily chelated with cations to form insoluble compounds. Although many soils are rich in phosphorus, the content of available phosphorus in the soil for plant absorption and utilization is very low, which makes the utilization efficiency of phosphorus fertilizer much lower than that of nitrogen and potassium [9]. Phosphorus deficiency and low utilization rate are common problems in most cultivated land in China, but long-term excessive application of phosphorus fertilizer causes more serious agro-ecological disasters [10]. To address this issue, an important way is to study the crop gene responsible for the efficient phosphorus uptake, thereby improving the utilization rate of phosphorus in soil [11]. At present, five families of plant phosphate transporters have been found, including PHT1, PHT2, PHT3, PHO1 and PHO2. The phosphate transporter PHT1 family was popularly studied and PT6 belongs to the PHT1 protein family. PT6 is a high affinity phosphate transporter with dual functions of phosphate absorption and transport [12–15]. *PT6* gene is specifically expressed in the root and the phosphorous-deficient parts of the plant ground, and it plays a role in the absorption and transport of phosphorus [16–18]. The main cultivation area of hulless barley is located in the plateau with mostly barren land, in which the ecological environment problems were caused by an excessive fertilizer use. Furthermore, the Qinghai-Tibet Plateau is the source of several important river systems in China, thus making the ecosystems more sensitive and vulnerable. The agricultural ecological environment protection of the Qinghai-Tibet Plateau is conferred a special ecological status in the whole China, as it is directly related to the economic development of the Qinghai-Tibet Plateau. In recent years, a strategy has been adopted to reduce the amount of chemical fertilizers and increase the efficiency of agricultural production for the economic development on the Qinghai-Tibet Plateau.

Cloning and studying the genes with properties of high efficiency absorption and utilization of phosphorus in hulless barley is of great significance to avert the low phosphorus tolerance of hulless barley. The study of plant phosphate transporters will be helpful to improve the efficiency of phosphorus utilization and reduce the application of chemical fertilizer in crop cultivation, thereby reducing the agricultural production costs. It is of great significance to promote the development of ecological agriculture in the hulless barley cultivation area of China. Currently, there are very few studies on the genes related to phosphorus absorption and utilization in hulless barley. In the present study, the high affinity phosphate transporter gene *HvPT*6 was isolated from the hulless barley “Kunlun 14.” Bioinformatics software was used to analyze the gene structure, *cis*-acting elements in promoter region, protein physicochemical properties, phosphorylation sites, signal peptides, transmembrane structures, phosphorylation sites, and secondary and tertiary structures of HvPT6. Homologous protein amino acid sequences in other plants were aligned and a phylogenetic tree was constructed for analysis. In addition, the subcellular localization showed that HvPT6 was accumulated in cell membrane of *Nicotiana benthamiana* leaves. The expression of *HvPT6* in different hulless barley tissues, and the response to abiotic stresses or different plant hormone treatments was analyzed. Our results highlight the genes of high efficiency phosphorus absorption and utilization of hulless barley.

## 2 Materials and methods

### 2.1 Cloning of *HvPT6* cDNA and promoter region

According to the reported rice *OsPT6* sequence (XP 01564911.1; HORVU5Hr1G110220.4), the barley *HvPT*6 sequence was searched in the plant genome database Gramene (http://www.gramene.org/) for its genetic information [19,20]. The barley *HvPT*6 gene and the sequence of promoter regions was isolated from the barley variety “Kunlun 14” genomic DNA using a KOD-FX high-fidelity PCR enzyme. cDNA fragments of the *HvPT*6 were isolated according to primers forward, 5′ AAGGCTCGCGGCCATGGCGCGC 3′ and reverse, 5′ CGTACGGCGACGTCTCACA 3′. The promoter region of the *HvPT*6 were isolated by degenerate primers forward, 5′ TCACCAAGGCACAAGAGGCA 3′ and reverse, 5′ TTTTAGGGTGGGACGAGCCG 3′. The extracted hulless barley DNA was used for PCR amplification. The procedures were as follows: 94°C for 2 min; 98°C 10 s, 56°C 30 s, 68°C 2 min, 35 cycles; 4°C. After the PCR reaction, 5 μL PCR product was subjected to a 1% agarose gel containing ethidium bromide and detected under ultraviolet gel imager. The PCR products were then cloned into the pEASYBlunt vector for sequencing.

### 2.2 Bioinformatics analysis

The gene structure of *HvPT*6 was predicted by using the GSDS 2.0 (http://gsds.cbi.pku.edu.cn/). The promoter elements were predicted using the PlantCARE (http://bioinformatics.psb.ugent.be/webtools/plantcare/html/). The physical and chemical properties of HvPT6 protein were predicted using the Protparam (https://web.expasy.org/protparam/). Phosphorylation sites were analyzed using kinasephos2 (http://kinasephos.mbc.nctu.edu.tw/index.php). The signal peptide was predicted using the SignalP 5.0 server (http://www.cbs.dtu.dk/services/SignalP/). Transmembrane topology was performed using the TMHMM-2.0 (http://www.cbs.dtu.dk/services/TMHMM-2.0/). Secondary structures were predicted using the SOPMA (https://npsa-prabi.ibcp.fr/cgi-bin/npsa_automat.pl?page=npsa_sopma.html). The tertiary structure was predicted using the AlphaFold2 (https://wemol.wecomput.com/ui/#/). The alignment of the nucleotide and protein sequences was performed using DNAMAN 7.0 and the phylogenetic analysis was done using the MEGA 7.0.

### 2.3 Expression profile analysis of *HvPT6*

To analyze the *HvPT*6 expression level in hulless barley “Kunlun 14,” the flag leaves, roots, stalks, grains and internodes at the grain filling stage were harvested for RNA extraction. To analyze the expression of *HvPT*6 induced by plant hormones and abiotic stresses, the five leaf stage of hulless barley “Kunlun 14” were treated with low phosphorous stress, PEG-6000 simulated drought stress, NaCl salt stress, and plant hormones like abscisic acid (ABA), methyl jasmonate (MeJA), cytokinin (6-BA), auxin (NAA), gibberellin (GA_3_) and salicylic acid (SA), and then the leaves were harvested for RNA extraction.

Hulless barley “Kunlun 14” seeds were soaked by 84 NaClO disinfectant for 6 min and washed with water five times, and then put into a petri dish with a filter paper for germination under room temperature. After 5 days, the consistent growing 50 seedlings were fixed in foam board and cultured in a plastic box (600 mm × 500 mm × 160 mm) using 20L Hoagland’s liquid medium. Air was pumped into the medium for 24 h using an air pump, and the culture medium was changed every 3 days with 1 mol L^−1^ KOH solution to stabilize the pH 7.2. The hulless barley seedlings with the five leaf stage were treated with low phosphorous stress (10 μmol L^−1^ KH_2_PO_4_ with phosphorous free Hoagland’s medium) according to Nadira et al. [21], 30% PEG-6000 simulated the drought stress according to the method of Zheng et al. [22], and 200 mmol L^−1^ NaCl was used to induce salt stress according to Duan et al. [23]. The plant hormone treatments included 100 µM of ABA, MeJA, 6-BA, NAA, GA_3_ and 2.5 mM of SA solution containing 0.1% Tween-20, respectively, according to An et al. [24]. Subsequently, three leaves and roots of the treated plants were harvested at the time points of 0 h (no treatment control), 6, 12, 24, 48, 72 and 96 h after treatment. All the samples were analyzed consisting of at least three biological replicates.

RNA from the leaves, roots, stalks and internode was extracted using the TransGen Transzol Up Plus kit. The polysaccharide polyphenol plant RNA extraction kit (Tiangen Biochemical Technology Co., Ltd) was used to extract the RNA from the grains. The cDNA was synthesized using the first-strand cDNA synthesis super mix kit (Transgen Biotech, Catalog No. AE301-02). The pair of specific primers of *HvPT*6 were as follows: forward, CGCGCTCACCTTCTTCTTCG and reverse, TGTACCCGTGGTCCACCTTG. The primers of reference gene 18SrRNA was as follows: forward, CGGCTACCACATCCAAGGAA and reverse, GCTGGAATTACCGCGGCT. The reaction system consisted of 1.0 μL primers, 2.0 μL cDNA, 10 μL Thunderbird SYBR qPCR Mix and 6.0 μL ddH_2_O. The formula 2^−∆∆Ct^ was used for qRT-PCR analysis and each reaction was repeated for three times.

### 2.4 Subcellular localization analysis of the HvPT6

The primers for vector construction follows, forward, 5′ GCTCTAGAAAGGCTCGCGGCCATGGCGCGC 3′ and reverse, 5′ CGGGTACCCACGGGCACCGTCCTGGCGT 3′, and the PCR fragment was cloned into the plant expression vector pBI221-GFP at the Xba Ⅰ and KpnI sites for subcellular localization. The final construction pBI221:HvPT6-GFP was transformed into *Agrobacterium* strain GV3101, which was injected into 40 days old tobacco leaves. After 48 h infiltration, the green fluorescent protein (GFP) fluorescence signal of HvPT6 was observed using the laser confocal scanning microscope (Nikon, C2-ER). The pBI221-GFP was transformed into tobacco leaves as a positive control.

### 2.5 Transformation of *Arabidopsis* and identification of transgenic plants

To generate the *HvPT*6-overexpressing transgenic *Arabidopsis* lines, the full-length cDNA of the *HvPT*6 was amplified and cloned into a binary vector pBI221 after the CaMV35S promoter, using the One Step Cloning Kit (Vazyme Biotech, Nanjing, China) according to the manufacturer’s protocol. The positive vector was transiently transferred to *Agrobacterium* strain GV3101 using the freeze–thaw method. Next, the *Agrobacterium* strain GV3101 containing pBI221:*HvPT*6 vector was introduced into *Arabidopsis* using the floral dip method for *Arabidopsis* transformation. Further, the seeds were harvested from the transformed plants and sterilized with 75% ethanol for 30 s, followed by 20% hypochlorite for 20 min. These seeds were selected on Basta (20 mg L^−1^) medium for the positive transgenic seedlings. DNA from the resistant plants was extracted for PCR with *HvPT*6-specific primers to check for the target gene insertion. Homozygous T3 or T4 seeds were used for further research.

### 2.6 Phosphorus starvation treatment and plant index measurements

Transgenic seedlings were grown on 1/2 MS medium containing 0.8% agar, 1% sucrose and 50 mg L^−1^ kanamycin. After germination, the wild-type and transgenic *Arabidopsis* seedlings of similar size were selected and transferred to MS medium with 10, 50, 100 and 625 mM KH_2_PO_4_, respectively. Subsequently, these plants were observed for the phenotype at 10 days and the related indexes were measured. The roots were photographed under the scanner (Epson, Expression 11000XL, Japan) and measured by a straightedge. The number of lateral roots was counted using the ImageJ program and plants were weighed using an analytical balance. Data were recorded from five individual plants from each treatment. For measurement of the anthocyanin content, 2-week-old plants were grown in MS medium with different Pi levels and 0.05 g of leaves were exposed to 500 μL 1% HCl–methanol mixture at 4°C overnight. Then, 300 µL of ddH_2_O_2_ and 300 μL of trichloromethane were added and these tubes were centrifuged at 14,000 rpm for 15 min. Finally, the upper water phase was detected by microplate reader at 530 and 657 nm for determination of absorbance value. Anthocyanin concentration was calculated using the formula: A530-0.33A657. For measurement of phosphorus content, the phosphorus content was determined using the phosphorus content detection kit (Solarbio, China). Each experiment consisted of three biological replicates.

## 3 Results

### 3.1 Cloning and gene structure analysis

**Figure A1:**
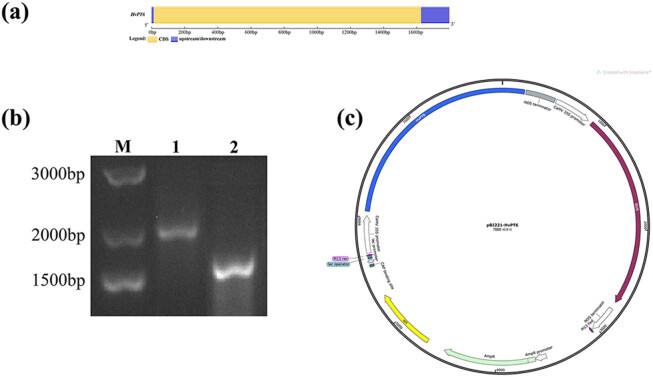
Nucleotide sequence amplification of *HvPT6*: (a) model of *HvPT6v* gene structure and (b) amplification of *HvPT6* by PCR. M, Marker; 1, ORF of *HvPT6*; 2, promoter fragments of *HvPT6*.

Complementary DNA (cDNA) containing an open reading frame (ORF) orthologous to the rice STP family member Os*PT6,* designated *HvPT6* was isolated from a cDNA library constructed from hulless barley “Kunlun 14”. *HvPT6* cDNA with a predicted ORF of 1,641 bp and a 2,199 bp promoter region fragment were amplified by PCR (Figure A1). The amplified fragments were linked into the vector pASY-Blunt Cloning vector and sequenced with M13 primers to obtain the gene and the promoter region fragments of hulless barley *HvPT6*. The gene structure of *HvPT6* (HORVU5HR1G1102204) was generated by using the input Gene Structure Display 2.0 software by sequencing, and the transcript corresponding to *HvPT*6 did not contain introns (Figure A1).

### 3.2 Prediction of elements of the *HvPT6* promoter region

The cloned promoter region sequence of the *HvPT6* gene was analyzed by the PlantCARE software. Three MBS elements were found in the promoter region, which could be related to drought inducibility (Table 1). In addition, there were a lot of *cis*-active elements corresponding to the response to the plant hormones such as SA, JA, ABA and GA (Table 1). Specifically, three ABRE elements were involved in the ABA responsiveness and two TCA elements were involved in SA responsiveness.

**Table 1:** Analysis of *cis*-acting elements in *HvPT*6 promoter region

**Element**	**Motif**	**Function**	**Number**
ABRE	ACGTG; GCAACGTGTC; AACCCG	Cis-acting element involved in the abscisic acid responsiveness	3
CAAT-box	CAAT; CCAAT; CAAAT	Common *cis*-acting element in promoter and enhancer regions	40
CGTCA-motif	CGTCA	Cis-acting regulatory element involved in the MeJA-responsiveness	1
G-box	TACGTG; CACGTG;	Cis-acting regulatory element involved in light responsiveness	2
GC-motif	CCCCCG	Enhancer-like element involved in anoxic specific inducibility	1
LTR	CCGAAA	Cis-acting element involved in low-temperature responsiveness	1
MBS	CAACTG	MYB binding site involved in drought-inducibility	3
O2-site	GATGATGTGG	Cis-acting regulatory element involved in zein metabolism regulation	1
P-box	CCTTTTG	Gibberellin-responsive element	2
Sp1	GGGCGG	Light responsive element	3
TATA-box	TATACA; TATA; TATAA; ATATAA;	Core promoter element around −30 of transcription start	11
TCA-element	CCATCTTTTT	Cis-acting element involved in salicylic acid responsiveness	2
TGACG-motif	TGACG	Cis-acting regulatory element involved in the MeJA-responsiveness	1
AE-box	AGAAACAA	Part of a module for light response	1
GT1-motif	GGTTAA	Light responsive element	1
Circadian	CAAAGATATC	Cis-acting regulatory element involved in circadian control	1

### 3.3 Physicochemical properties and structure analysis of the HvPT6 protein

**Figure A2:**
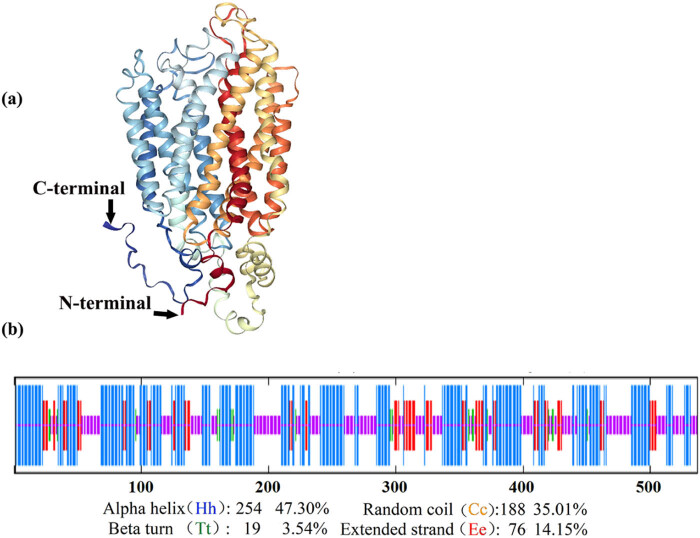
Structure analysis of *HvPT6*: (a) 3D structure prediction diagram and (b) structure analyzed by the SOPMA software.

**Figure A3:**
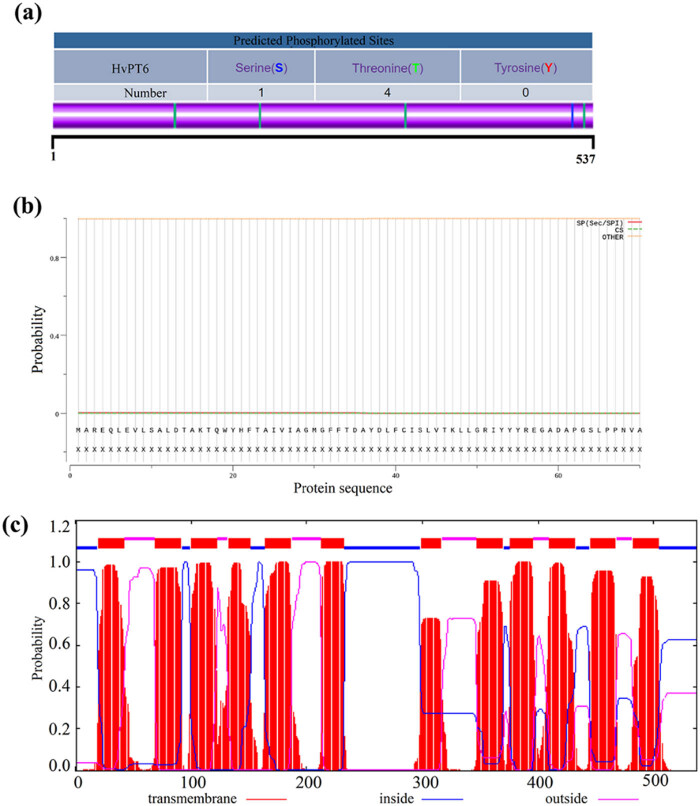
Bioinformatics analysis of *HvPT6*: (a) phosphorylation site prediction map, (b) signal peptide prediction map and (c) transmembrane domain prediction of HvPT6.

The physicochemical properties of the hulless barley HvPT6 protein were analyzed by the ProtParam software (Table 2). The protein was composed of 537 amino acids and was a hydrophobic stable protein. It was found that the α-helix, β-turn, extended strand and random coil accounted for 47.30, 3.54, 14.15 and 35.01%, respectively, of the total amino acids in the secondary structure of HvPT6 protein using SOPMA software (Figure A2). Three-dimensional (3D) structure prediction of HvPT6 showed that the alpha helical and random coil accounted for most of the structure area and the rest of the structures were scattered among them (Figure A2b). The tertiary structure prediction results were basically consistent with the secondary structures. The amino acid phosphorylation sites of HvPT6 protein contained five phosphorylation sites, including 1 serine (Ser), 4 threonine (Thr), and no tyrosine residues (Figure A3a). Signal peptides analysis using the SignalP-5.0 software showed that HvPT6 did not have the signal peptide (Figure A3b). Twelve transmembrane structures from HvPT6 protein were confirmed by TMHMM Server V.2.0 software (Figure A3c).

**Table 2:** Physical and chemical properties of HvPT6 protein

**Protein name**	**Molecular weight**	**Total number of atoms**	**Formula**	**GRAVY**	**Theoretical pI**	**Instability index (II)**	**Aliphatic index**
HvPT6	58387.63	8,186	C_2691_H_4064_N_682_O_722_S_27_	0.362	7.98	34.19	87.34

### 3.4 Amino acid sequence alignment and phylogenetic tree construction

**Figure 1:**
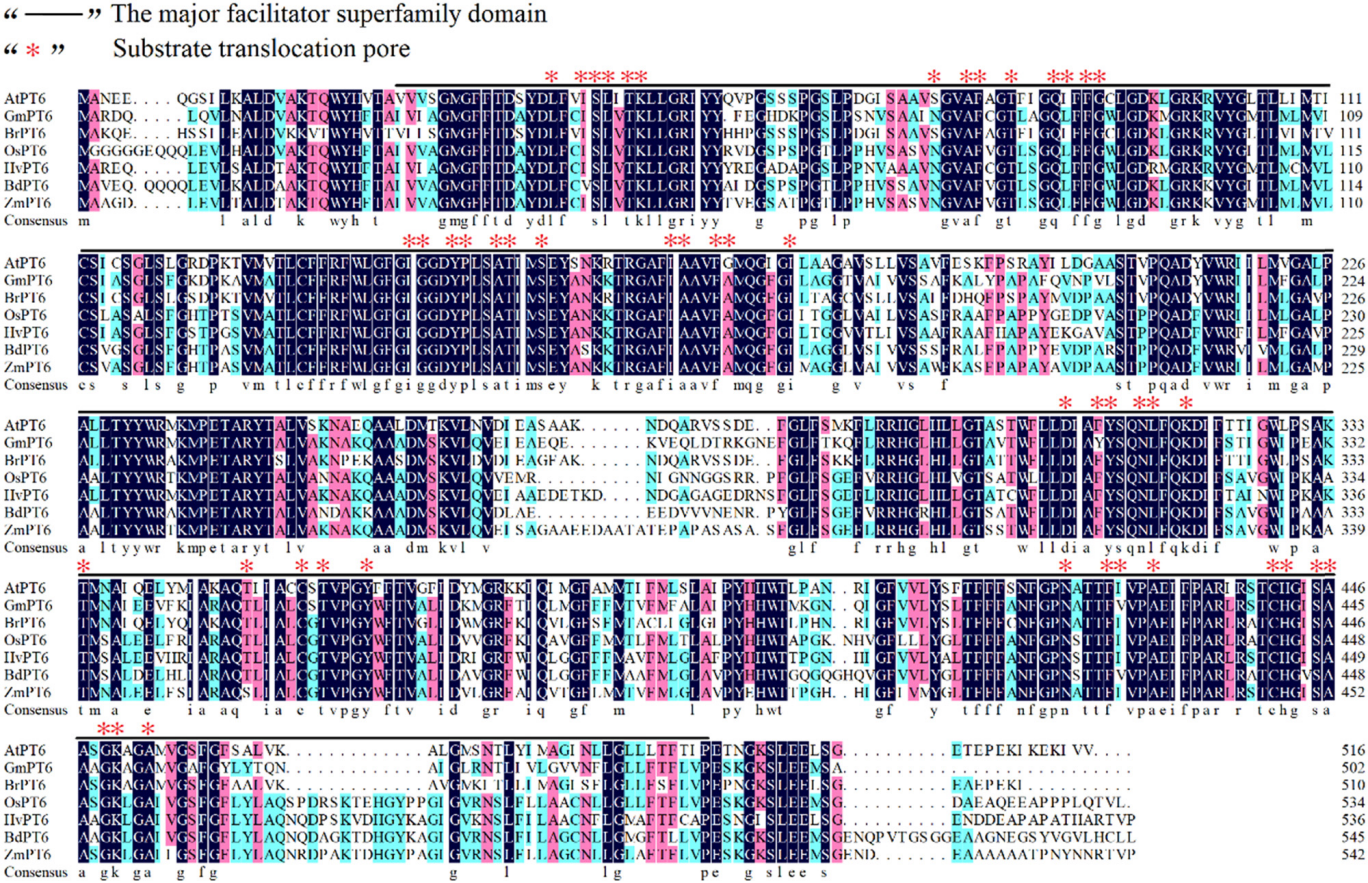
Multiple sequence alignment of HvPT6 with its homologs from other species. Protein sequence alignments between HvPT6 and its homologs from other species were performed by DNAMAN software (Version 6). Asterisks ✽ represent substrate translocation sites and the solid line indicates the major facilitator superfamily domain.

**Figure 2:**
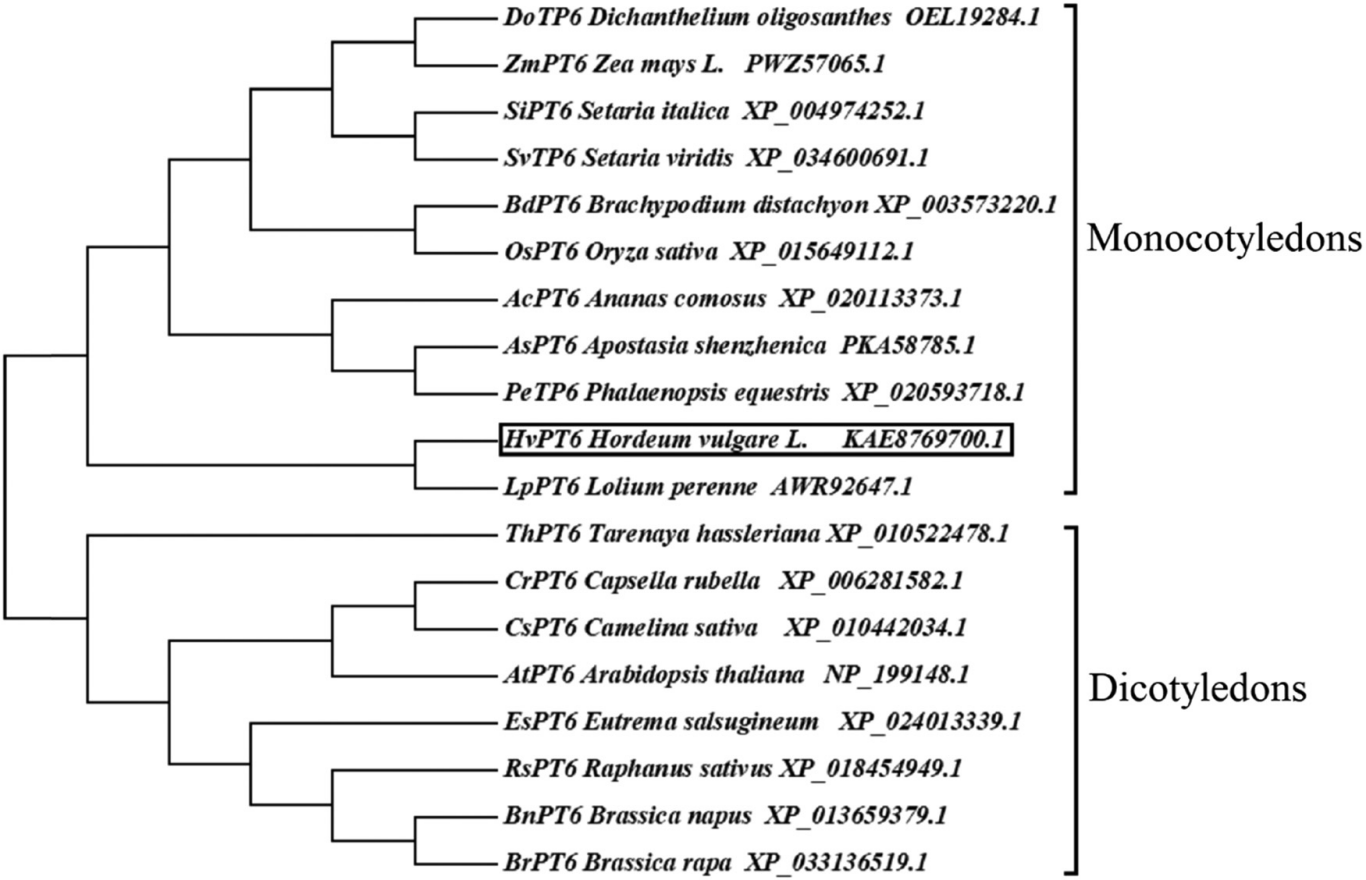
Phylogenetic analysis of HvPT6 with its homologs from other species. Phylogenetic analysis of phosphate transporter from dicotyledon and monocotyledon plants using MEGA5. Branches are labeled with GenBank accession numbers and the organisms. The box represents HvPT6.

The amino acid sequences of HvPT6 and its homologs from *Arabidopsis thaliana*, *Brachypodium distachyon*, *Brassica napus*, *Zea mays* L., *Oryza sativa*, *Brassica rapa* and *Glycine max* (Linn.) *Merr*. were extracted from the NCBI database and analyzed by DNAMAN 7.0. The results showed that AtPT6, BdPT6, BnPT6, ZmPT6, and OsPT6 had high similarity with HvPT6. All these proteins possessed the major facilitator superfamily domain (Figure 1). The homologous protein sequences from hulless barley and 18 other plants were analyzed and their phylogenetic tree was constructed using MEGA7. The results showed that the phylogenetic tree was mainly divided into monocotyledon and dicotyledon, among which HvPT6 belonged to the branch of monocotyledon, and it had the highest similarity to LpPT6 from rye grass (Figure 2).

### 3.5 Expression pattern analysis of *HvPT6*

**Figure 3:**
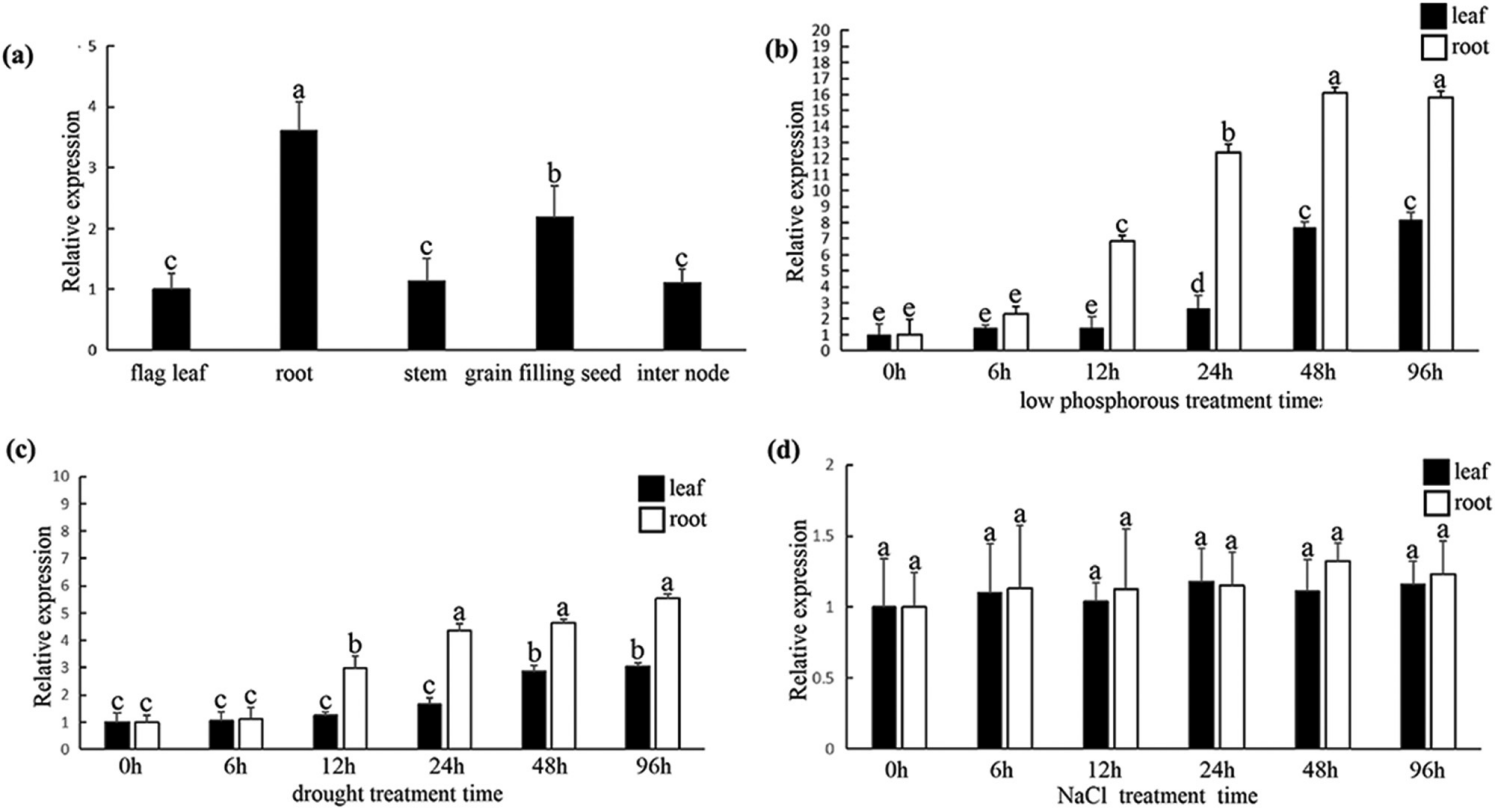
Expression patterns of *HvPT6* in different barley tissues and in response to abiotic stresses: (a) expression of *HvPT6* in root, stem, flag leaves, inter node and seed, (b–d) expression patterns of HvPT6 in response to phosphate starvation, drought and salt stress, respectively.

The expression pattern of *HvPT6* was first analyzed in different barley tissues by qPCR. *HvPT6* was the highest in the roots, followed by grains. However, there was little difference in the expression levels in leaves, stems and internodes (Figure 3a). To determine whether *HvPT6* possessed a role in response to stresses, the expression of *HvPT6* was analyzed in the leaves and roots of hulless barley under phosphate starvation. *HvPT6* in the leaves increased significantly after 24 h, and but *HvPT6* increased significantly after 6 h in the roots. After 48 h of low phosphorus treatment, the expression of *HvPT6* tended to be stable in the leaves and roots (Figure 3b). PEG-6000 simulated the drought stress treatment and proved that *HvPT6* could be also induced in the leaves and roots of hulless barley under drought stress (Figure 3c), but the expression of *HvPT6* did not change significantly under NaCl salt stress (Figure 3d).

**Figure 4:**
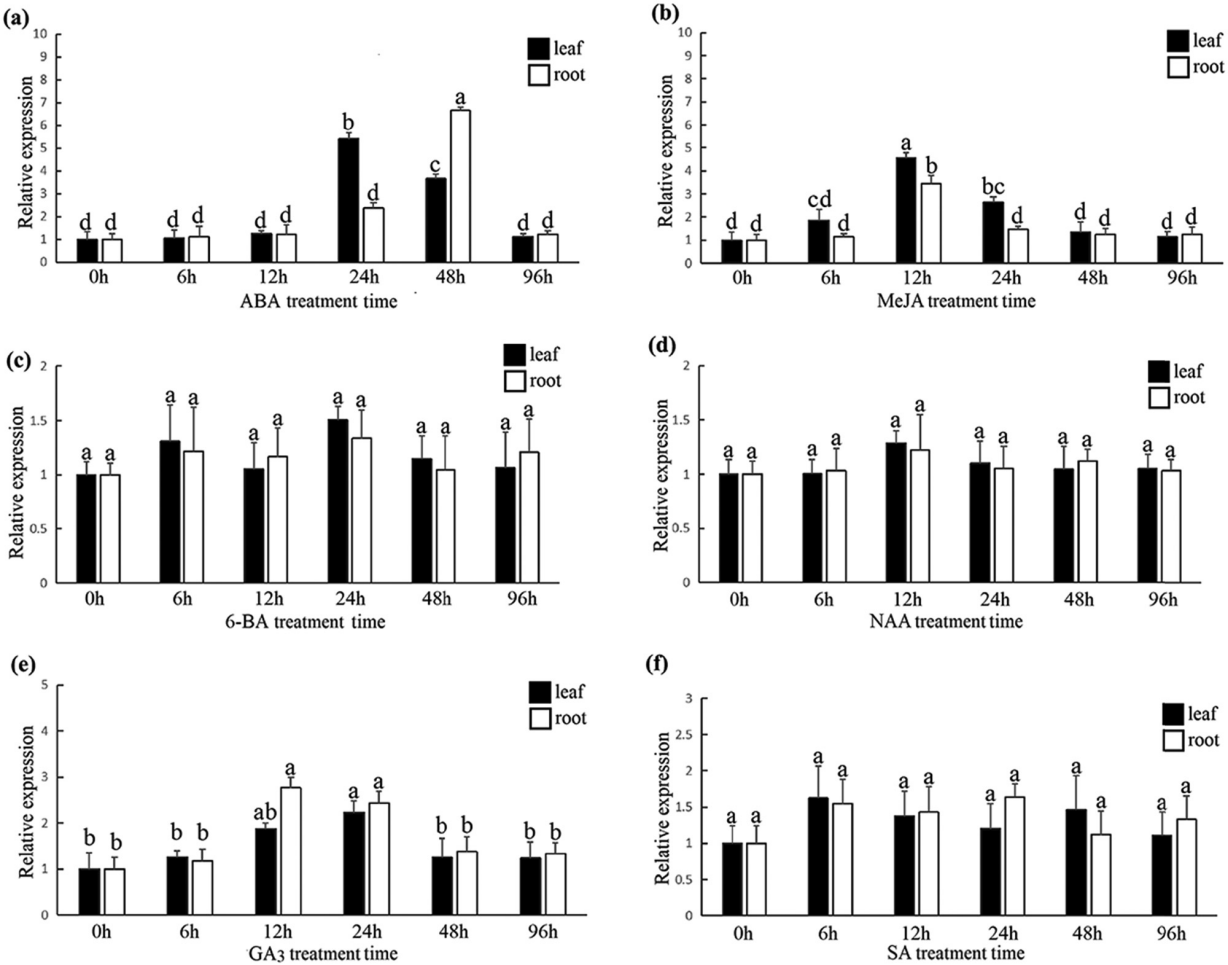
Expression profile of *HvPT6* in response to exogenous hormones (a–f) ABA, MeJA, 6-BA, NAA, GA_3_, SA.

In view of the many *cis*-acting elements related to plant hormones in *HvPT6* promoter region, we tested whether *HvPT6* was induced by plant hormones in the leaves and roots. Interestingly, different plant hormone treatment assays showed that the expression of *HvPT6* was higher in the leaves and roots of hulless barley with ABA, MeJA and GA_3_ treatment (Figure 4). On the other hand, *HvPT6* was not induced in the leaves and roots of hulless barley treated with 6-BA, NAA and SA (Figure 4).

### 3.6 Subcellular localization of HvPT6

**Figure 5:**
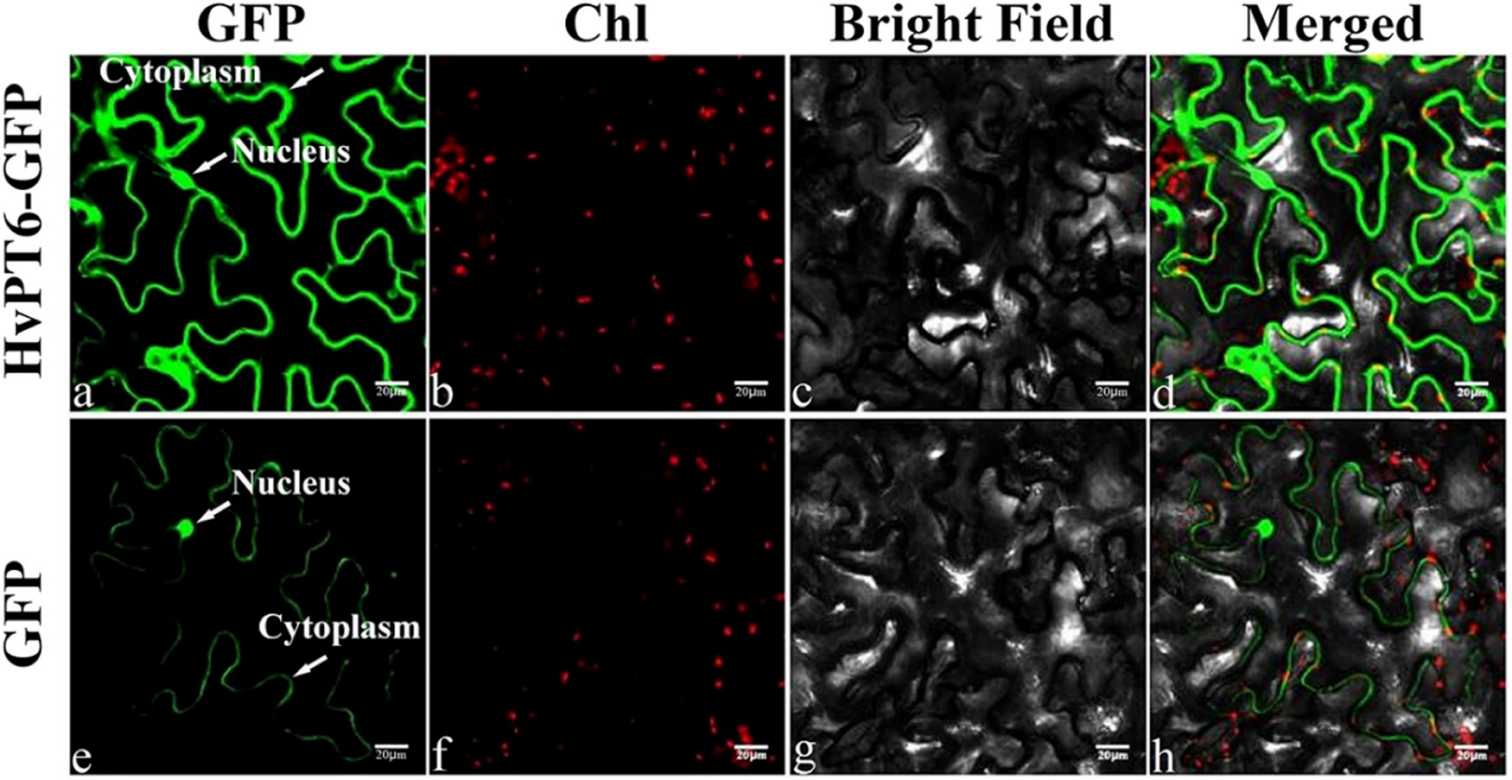
Subcellular localization of HvPT6 in *N*. *benthamiana* leaves. Leaf tissues of *N. benthamiana* transiently co-expressing the HvPT6-GFP or GFP alone were examined by Laser Scanning Confocal Microscopy (Nikon C2-ER). Green fluorescent protein (GFP HvPT6 fusion protein (a–d) or GFP protein (e–h) was transiently expressed in *N*. *benthamiana* leaves. (a and e) GFP signal, (b and f) chlorophyll fluorescence, (c and g) bright field, (d and h) merged photos. Bar = 20 µm.

Because HvPT6 is a phosphate transporter, we speculated that it may localize on the plant membrane. To understand the localization in plant, HvPT6: GFP protein in tobacco leaves was transiently expressed by using transient expression of *Agrobacterium*. Only the GFP signal was observed in cytoplasm and nucleus of *N*. *benthamiana* (Figure 5). But the fluorescence signal of HvPT6: GFP protein was mainly accumulated in the cell membrane of *N*. *benthamiana* (Figure 5), indicating that HvPT6 is a membrane protein.

### 3.7 Overexpressing *HvPT6* for improved plant tolerance to low phosphorus treatment

**Figure 6:**
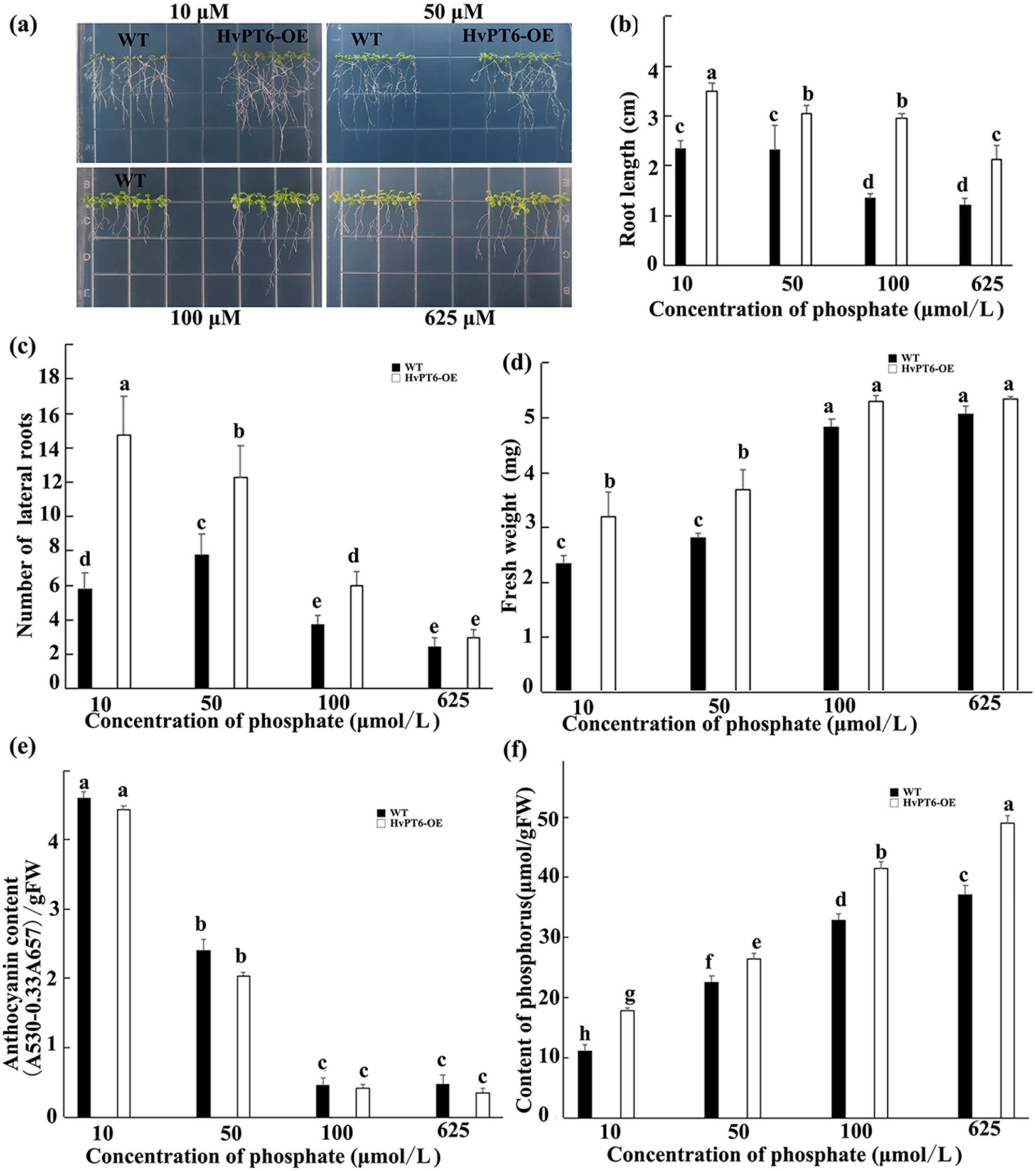
Overexpression of *HvPT6* enhances tolerance to phosphate starvation in *Arabidopsis*. (a) Phenotype of WT and *HvPT6*-overexpressing transgenic *Arabidopsis* seedings under 10, 50, 100 and 625 μM phosphate treatments. (b) Root length, (c) total number of lateral roots per plant, (d) fresh weight, (e) anthocyanin content and (f) total phosphorus were determined in WT and transgenic Arabidopsis plants under 10, 50, 100 and 625 μM phosphate treatments. Data represented as mean of three replicates with errors bars indicating SD at *P* ≤ 0.05.

To investigate the role of *HvPT6* in response and adaptation to low phosphorus, we generated the *HvPT6*-overexpressing transgenic *Arabidopsis*. The transgenic plant and the WT were exposed to MS medium with different concentrations of Pi, and it was observed that the vegetative growth of transgenic *Arabidopsis* was not inhibited under low Pi condition (10 μM) compared to normal Pi conditions (625 μM) (Figure 6a). The results of root length of the *HvPT6*-overexpressing *Arabidopsis* also showed a better growth (1.5–2-fold length) compared with WT under low Pi concentration (Figure 6b). The number of lateral roots of WT and transgenic plants grown in MS medium plates under different Pi concentrations was also assessed. Low phosphorus (10 μM) treatment significantly promoted the number of lateral roots of transgenic plants (about 14), compared with that of WT (about 6) (Figure 6c). The results of the fresh weight also showed a better growth in the *HvPT6*-overexpressing *Arabidopsis* (3.3 mg) compared with WT (2.3 mg) under 10 μM Pi treatment (Figure 6d). However, non-significant differences in the anthocyanin contents of *HvPT6*-overexpressing plant indicated that the transgenic plants did not affect the adaptability of *Arabidopsis* to phosphate starvation (Figure 6e). Finally, the concentration of total phosphorus in transgenic Arabidopsis was detected and it was found to be significantly higher (1.5-folds) compared with WT under different Pi levels (Figure 6f). These results indicated that overexpressing the *HvPT6* gene improved the plant tolerance to phosphate starvation and made the plants better adapt to low Pi condition.

## 4 Discussion

Phosphate is one of the important nutrient elements involved in plant growth and metabolism. Phosphate transporters are responsible for the uptake and transport of phosphorus in plants, and are induced by low phosphate in roots. Both high and low affinity phosphate uptake and transport systems were found in plants, and most of the Pht1 family phosphate transporters belong to the high-affinity phosphate uptake and transport systems [25]. In *A. thaliana*, AtPHT1;1 and AtPHT1;4 were identified for the acquisition of phosphate from both low and high phosphate environments [26]. In rice, OsPT8 was reported to be constitutively expressed transporter with the function of phosphate homeostasis [27]. Although a growing number of PHT genes in different plants have been identified, their molecular functions are not clearly elucidated. In this study, we focused on PHT gene *HvPT6*, an important member of the PHT1 family in rice and other model plants, while there are few reports on other plants [28].

Many studies have shown that PT6 protein is only localized on the cell membrane [13,29], which needs further research. We also found that the HvPT6 from hulless barley and LpPT6 from ryegrass were the most closely related, as proved from the findings of the phylogenetic tree. Furthermore, HvPT6 protein and its homologues in different plants are highly conserved with six N-terminal transmembrane regions and six C-terminal transmembrane regions, separated by hydrophilic regions [15]. The PHT1 phosphate transporter family has different roles in different plants. Zhao et al. studied the spatiotemporal expression characteristics of 13 genes (*OsPT1*-*OsPT13*) in the rice phosphate transporter family PHT1, and found that these 13 genes had difference in the spatiotemporal expressions, among which *OsPT6* was strongly induced in roots, rhizome juncture, leaves, anthers and seedlings under phosphorus deficiency [30]. Similarly, the expression of *HvPT6* was higher in grain and root than in leaves, stalks and internodes, which could be related to its function on phosphorus uptake in hulless barley roots, which are a major site of Pi uptake from the soil. Ai et al. found that the expression of *OsPT2* and *OsPT6* gene from rice gene mainly accumulated in the young taproot, lateral root and rhizome under the low phosphorus conditions [15]. But the role of HvPT6 in phosphorus remobilization in the leaves remains unclear. In *A. thaliana*, AtPHT1;5 was involved in mobilizing Pi from phosphorus source to sink organs in accordance with developmental cues and phosphorus status. *OsPT2* is mainly responsible for transferring the phosphate to the ground part, but *OsPT6* has a role in the absorption and transferation [31]. Wang et al. found that *OsPT6* was highly expressed in the underground part in high-yielding rice varieties “Wu yu jing 7” materials than the ground part, and *OsPT6* was induced by a phosphorus deficiency [32]. The phosphorus content of *OsPT6* overexpressing plant was also higher than the wild type.

Many phosphate transporters were induced by several phytohormone, biotic and abiotic stresses. Liu et al. studied the expression patterns of *OsPT6* in the rice leaves treated with different plant hormones and showed that *OsPT6* was induced by ABA and 2,4-D, while it could not be inhibited by NAA. Further, KT and GA_3_ had no effect on the expression of *OsPT6* [33]. It was found that *HvPT6* was induced by drought, ABA, JA, MeJA and GA_3_ in the leaves and roots of hulless barley, indicating that *HvPT6* played a role in the abiotic stress. OsPT11 and OsPT13 were reported to be involved in the arbuscular mycorrhizal fungal symbiosis [34]. TaPT29-6A-silenced lines reduced the levels of AM fungal colonization and arbuscules, but increased susceptibility to biotrophic and hemi-biotrophic pathogens, indicating that TaPT29-6A was not only essential for the AM symbiosis, but also in the plant immunity [35]. To better understand the function of *HvPT6* on phosphate absorption, experiments revealed that the overexpression of *HvPT6* improved the tolerance to low phosphorus in transgenic plants than the control. The inorganic Pi in soil solution lower than 10 μM could fit with the high-affinity role for these transporters in the root [36]. Overexpression of *HvPT6* significantly enhanced the uptake ability of phosphorous in a solution culture with 10 μM Pi, which provides additional evidence for the high-affinity properties. Thus, it is meaningful to study the underlying regulatory molecular mechanisms of *HvPT6* in greater detail.

According to the analyses of gene and protein structure, expression pattern and function of HvPT6, the results showed that HvPT6 gene and protein structure had typical characteristics of PHT1 phosphate transport family. Its expression was induced by low phosphorus and plant hormones, indicating that it may also be involved in the regulation of low phosphorus stress in hulless barley. The transfer of HvPT6 into Arabidopsis showed that it could significantly improve the tolerance of transgenic plants to low phosphorus stress, which indicated that HvPT6 had potential application value in improving the tolerance of plants to low phosphorus tolerance by molecular biology. This study will provide a reference for research on the phosphate transporter family of hulless barley and the use of molecular biology to breed hulless barley varieties with efficient phosphorus uptake.
